# Assessing distinguishable social skills in medical admission: does construct-driven development solve validity issues of situational judgment tests?

**DOI:** 10.1186/s12909-022-03305-x

**Published:** 2022-04-20

**Authors:** Ina Mielke, Simon M. Breil, Dorothee Amelung, Lia Espe, Mirjana Knorr

**Affiliations:** 1 Department of Biochemistry and Molecular Cell Biology, University Medical Center Hamburg-Eppendorf, N30, Martinistraße 52, 20246 Hamburg, Germany; 2 Department of Psychology, University of Münster, Münster, Germany; 3 Office of Student Affairs, Heidelberg University Hospital, Heidelberg, Germany; 4 Division of Medical Teaching and Education Research, Göttingen University Medical Center, Göttingen, Germany

**Keywords:** Situational judgment test, Social skills, Medical school admission, Test development

## Abstract

**Background:**

Social skills are important for future physicians and are therefore increasingly considered in selection processes. One economic assessment method from which different social skills can be inferred are Situational Judgment Tests (SJTs) in which applicants are asked to rate behavioral responses in context-relevant situations. However, traditional SJTs have so far failed to distinctively measure specified constructs. To address this shortcoming in the medical admission context, we applied a construct-driven approach of SJT development in which test development was deductively guided by agency and communion as target constructs.

**Method:**

The final version of the construct-driven SJT includes 15 items per construct with three behavioral responses. Medical school applicants (*N* = 1527) completed the construct-driven SJT, a traditional SJT, and an aptitude test under high-stakes condition as part of their application. A subsample (*N* = 575) participated in a subsequent voluntary online study with self-report measures of personality and past behavior.

**Results:**

The proposed two-factor structure and internal consistency of the construct-driven SJT was confirmed. Communal SJT scores were positively associated with self-reported communal personality and communal behavior, yet effects were smaller than expected. Findings for agentic SJT scores were mixed with positive small associations to self-reported agentic personality scores and agentic behavior but unexpected negative relations to communal self-reported measures.

**Conclusions:**

Results suggest that construct-driven SJTs might overcome validity limitations of traditional SJTs, although their implementation is challenging. Despite first indicators of validity, future research needs to address practical points of application in high-stakes settings, inclusion of other constructs, and especially prediction of actual behavior before the application of construct-driven SJTs for selection purposes in medical admission can be recommended.

**Supplementary Information:**

The online version contains supplementary material available at 10.1186/s12909-022-03305-x.

## Background

Good physicians are not only characterized by clinical skills and knowledge, but also by social skills [1–3]. In particular, physicians need different social skills that allow them to function effectively across a wide array of different interpersonal situations. For example, when faced with a colleague’s conflicting opinion, a physician is expected to clearly argument for his or her professional view (i.e.*, agentic skill*), whereas when faced with a despondent patient, compassion and emotional support (i.e.*, communal skill*) is expected. That is, social skills describe how capable someone is of doing something in a given situation [4].

The importance of social skills is already acknowledged in many admission processes to medical schools, where they are considered as entry criteria in addition to cognitive abilities [5, 6]. One method that is discussed as an economic way to assess social skills of the typically high number of applicants to medical schools is a Situational Judgment Test (SJT [7–9]). SJTs provide applicants with a hypothetical situation and a range of behavioral response options, which need to be rated, ranked, or selected according to how applicants perceive their effectiveness or anticipate them to be in line with their own behavior in that situation. Individual differences are then assessed by aggregating behavioral responses across different interpersonal situations.

SJTs are well-known for their ability to predict meaningful outcomes like actual job performance [10–17]. However, there is also a number of unresolved issues including a lack of clarity on the SJT’s construct validity, i.e., what they actually measure. In fact, even though many SJTs aim to assess distinct constructs, a general higher order factor usually emerges which is difficult to interpret (e.g., a general ability to answer SJTs [11, 18–20]). Thus, it remains unclear whether SJTs measure intended constructs for the medical context and whether they therefore contribute to the selection of favorable applicants. We argue here that problems in interpreting SJT scores mainly originate from the development process. Typically, SJT items are based on critical incidents within the medical context and reflect a complex interplay of multiple constructs that are difficult to disentangle and define (we refer to SJTs developed in this way as “traditional SJTs”; see [21]). An alternative to SJT development that fosters interpretability is the construct-driven approach [18, 21, 22], which has been shown to yield improved results with regard to construct validity while retaining criterion validity in different contexts [23–25]. Tiffin et al. (2020) outlined that construct-driven SJTs might improve interpretability of SJT scores in medical admission because they are likely to produce a clear and reliable factor structure. As they present less context-specific information than traditional SJTs, they might be especially suitable for the early stages of selection into medical training [8, 21]. Following this idea, a recent study introduced a construct-driven SJT for teacher selection that assesses emotional regulation of applicants and showed good psychometric properties [26]. However, to our knowledge, no study has so far examined the application of construct-driven SJTs in high-stakes medical admission.

### Development of construct-driven SJTs

The development of construct-driven SJTs is based on the theory of the predefined construct that determines how situations (i.e., the hypothetical situations applicants encounter) and behavioral responses (i.e., the hypothetical behaviors in the situations) need to be constructed. Based on trait-activation theory [27], situations should be designed to include triggers that evoke individual differences in behavioral responses relevant to the predefined construct. For instance, if one aims to assess how individuals can enforce a goal or opinion, situations may include interactions with contrasting professional opinions, which in turn should trigger interindividual differences in assertiveness. Behavioral responses should be designed to depict graded levels of the construct [18]. Following this example, one might include responses ranging from low (i.e., deflecting full responsibility to colleague with a contrasting opinion) to high assertive behavior (i.e., convincing the colleague with arguments). To form an overall construct score, evaluations of behavioral responses are usually aggregated across a large variety of situations reflecting one construct [22]. This way, a comprehensive construct assessment is ensured independent of single, highly specific situations. If multiple constructs are addressed within a construct-driven SJT, items should each uniquely measure one single construct to ensure a differentiated assessment [18, 22].

When selecting specific social skills for SJT development in medical admission, one must consider two aspects. First and foremost, the selected social skills should be *crucial for future physicians*. Here, a large array of taxonomies lists relevant skills [2, 28–30]. Secondly, for distinct assessment it is necessary that the selected social skills reflect *clearly distinguishable constructs* that actually emerge in interpersonal situations. To this end, the large number of different skills included in taxonomies presents a challenge, as many listed skills include similar and difficult to distinguish behaviors (e.g., empathy, compassion, emotional understanding). In turn, some mentioned skills are so vague that they manifest in different behaviors depending on the situations. Also, what is understood by the respective skills varies greatly depending on who is asked (e.g., communication skill, interpersonal skill). As a solution, we propose broader skill constructs that a) refer to clearly distinguishable individual differences and b) incorporate the most important aspects of previous taxonomies. A large number of studies suggests that different interpersonal capacities can be represented by two, well-established constructs: *Agency* (i.e., getting ahead in social situations) and *communion* (i.e., getting along in social situations [31, 32]). Herein, *agency skill* can be described as the capacity to proactively pursue (one’s own) goals and take responsibility in social situations. *Communion skill* describes the capacity to establish and maintain positive relations with others. These broader constructs, in turn, map to a variety of more specific and relevant skills from the aforementioned taxonomies which include *leadership*, *decision-making*, and *responsibility* (all agency), or *empathy*, *relationship building*, and *teamwork* (all communion). Combined, agency and communion are commonly described as forming the core of what constitutes a socially skilled person (i.e., achieving one’s own goals without harming the needs of others [33]). That is, in the best-case scenario we would select students who have both the capacity for agency and communion. In fact, research shows that agency and communion can be identified as general factors captured in assessment centers [34, 35] and that this strategy can be successfully implemented in the context of multiple mini-interviews for medical admission [36] or police service [37]. Thus, for the development of the present construct-driven SJT, we focused on agency and communion as the two broad social skills that are highly relevant for future physicians and have been shown to be distinctively measurable.

### Study aim

With the present study, we aim at testing whether construct validity issues of traditional SJTs in medical admission can be overcome by using a construct-driven SJT development approach. Specifically, we introduce a construct-driven SJT measuring agency and communion as distinguishable and clearly defined constructs. The SJT was applied in the high-stakes context of medical admission and a) dimensionality and reliability, b) discriminant and convergent validity with a traditional SJT, an aptitude test, and self-reported personality and past behavior were assessed. We expected moderate relations of *r* = .20 between our SJT agency scale and agentic personality or past behavior and, accordingly, between our SJT communion scale and communal personality or past behavior [38]. Relations to measures of the other constructs and to other scales of self-reported personality were expected to be insignificant (see Supplement D for detailed results). To be transparent in line with recent open science practices our hypotheses were preregistered. The preregistration together with the full data analysis code and a template for data request can be retrieved from the Open Science Framework project via https://osf.io/6579b/.

## Methods

### Participants and procedure

Participants were persons interested in studying undergraduate medical or dentistry studies at University Medical Center Hamburg-Eppendorf. No preselection based on cognitive or other criteria was done at this stage of admission. For their application to the study programs, participants completed the construct-driven SJT, the HAM-SJT, a traditionally developed SJT, and the HAM-Nat, an aptitude test which consisted of four subtests (one science and three logical reasoning subtests). All tests were machine-marked paper pencil tests conducted on-site and in the same order on one of three alternative test days. The construct-driven SJT and one of the reasoning tests were integrated for research purposes and did not inform admission decisions. However, participants did not know that only the HAM-SJT part was relevant for admission and thus completed both SJTs under the same assumptions. Out of 1688 test participants, *N* = 1527 (90%^1^) gave voluntary consent that their admission data could be analyzed for study purposes in exchange for a detailed test feedback. This sample consisted of 66% female applicants (31% male, 2% no answer) with a mean age of 21.29 years (range: 15 to 37, *SD* = 2.66). A few days upon admission test completion, all participants who gave their consent were invited to an online survey via e-mail. The online survey included personality tests and questions on past behavior. A subsample of *N* = 575 completed the survey on a voluntary basis in exchange for a feedback on their personality test results and the chance to win one of 100 gift cards for an online shop, each of which was worth 50€ 2.

### Measures

Descriptive information and reliability assessed as internal consistency for all measures are provided in Table 1.

**Table 1:** Descriptive statistics, reliability, and intercorrelations of situational judgment tests, science test, personality, and behavior

	*M (SD)*	*α*	1.	2.	3.	4.	5.	6.	7.
1. SJT Agency	2.03 (0.24)	.63	–						
2. SJT Communion	2.62 (0.20)	.70	**−.17**	–					
3. HAM-SJT	3.53 (0.14)	.67	**.08**	**.23**	–				
4. HAM-Nat Science Subtest	0.16 (1.10)	.90	**.10**	.03	.01	–			
5. Agentic Personality	0.00 (0.82)	.84	**.10**	−.01	.04	.03	–		
6. Communal Personality	0.00 (0.81)	.82	**−.14**	**.15**	.02	**−.15**	.05	–	
7. Agentic Behavior	2.71 (0.52)	.60	**.10**	−.07	.04	.01	**.55**	**.08**	**–**
8. Communal Behavior	3.55 (0.48)	.75	**−.10**	**.12**	−.01	**−.11**	**.10**	**.56**	**.21**

Bold coefficients are significant with *p < .05*. Reliabilities for HAM-SJT and HAM-Nat science subtest are the mean reliabilities across different test versions. SJT = Situational Judgment Test. *N* = 1527 for SJTs and science subtest and *N* = 575 for personality and behavior

#### Construct-driven SJT

**Fig. 1:**
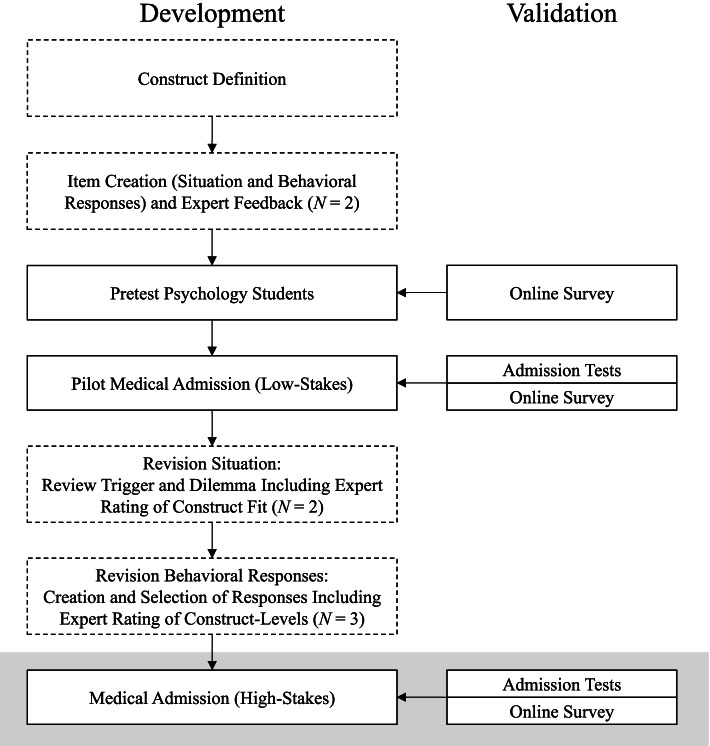
Development and validation process of the construct-driven Situational Judgement Test. Dotted boxes show steps of the revision process and solid boxes show SJT data collection. Numbers in parentheses refer to the number of raters per item. The grey background indicates data collections reported and analyzed in the current study. Detailed information on the pretest and pilot studies can be found in Supplement A

The SJT resulted from an iterative process involving all authors, other personality psychologists, and medical experts (see Fig. 1 for an overview and Supplement A for a detailed description and results from a pretest and pilot study), and followed recommendations from the literature on the development of construct-driven SJTs [18, 24]. In a first step, we formulated working definitions of agency and communion as well as triggering situations and high versus low construct-related behaviors 3. This framework guided the item writing and all following revision cycles. The initial item set consisted of 15 SJT items and items were selected based on similar ratings of construct experts and discriminatory power from a pretest study. As the resulting factor structure in a pilot sample showed some inconclusiveness, we decided on a final revision cycle. After we critically reviewed the construct-fit of all situations and edited them accordingly, two independent construct experts who had not been involved in earlier development steps were asked to match the situations to one of six constructs. We then checked whether each situation included an appropriate dilemma which would prevent participants from easily identifying the behavioral response exhibiting the most optimal expression of the intended construct while at the same time not reflecting expressions of the other construct (e.g., an agency item should not include a dilemma that triggers communion). In a next step, behavioral responses were adapted to the revised situation or newly developed. The resulting four to seven behavioral responses per situation were rated by three construct experts according to their construct level. Finally, three behavioral responses per item were selected based upon similar ratings and representation of the three different levels of the construct.

**Fig. 2:**
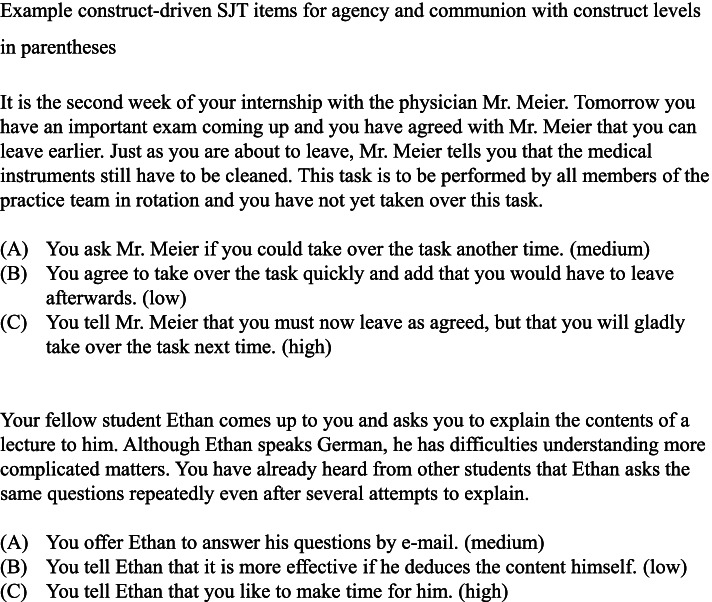
Example construct-driven Situational Judgement items for agency and communion with construct levels in parentheses

The final SJT includes 15 situations for agency and communion, respectively, with three behavioral responses per situation (see Fig. 2 for example items). All items are written in first person perspective and describe relevant situations from practical study training or education contexts, or situations with fellow students and friends. Participants are required to choose a behavioral response that would best describe their behavior in this situation, (i.e., behavioral tendency instruction [12]) and had a time limit of 25 min to complete all items in the current study. The selected responses were recoded according to the construct level into 1 (low), 2 (medium), or 3 (high). One score was obtained per construct by calculating the mean across the 15 related items.

#### HAM-SJT

The traditional HAM-SJT contained 22 situations and 86 behavioral responses (three to five per situation [40]). All items were derived from critical incidents and were not designed to match a single construct. They are written from the perspective of a third person and offer more context than items in the construct-driven SJT. Participants had to rate the appropriateness of each response for the situation at hand on a 4-point-scale from 1 (*very appropriate*) to 4 (*very inappropriate*). Participants’ ratings and expert ratings (which served as comparison criteria) were intra-individually *z*-standardized [41] and an average score of their squared difference across all behavioral responses was computed. The score was linearly transformed such that higher values reflect better performance.

#### HAM-Nat

In its current form, the HAM-Nat includes one science subtest and two subtests for reasoning ability. As the science subtest is best documented, we only report results on this subtest in the main part. The science subtest consisted of 60 multiple choice questions from the fields of biology, physics, chemistry, and mathematics [42, 43]. One in five possible answers was deemed correct. The final score is derived as a person ability parameter according to Item-Response-Theory with a higher score reflecting better performance [44].

#### Self-reported personality

We used three validated personality tests (BFI-2 [45]; IAL [46]; ISK [47]) that require participants to describe themselves on facets of agency and communion (see Supplement D for detailed results). As all three tests measure the same or very similar constructs and showed expected intercorrelations, we decided to aggregate them into composite scores of agentic and communal personality, respectively. Facet scores were first computed for each test separately, then *z*-standardized to account for different scales, and finally aggregated by using their mean value.

#### Self-reported past behavior

We included eight items asking participants to specifically focus on their behavior during the last six months [48]. Four items were designed to assess agentic behaviors (e.g., “[stand] up for your own needs”) and four items were designed to assess communal behaviors (e.g., “[show] understanding for others’ problems”; see Supplement E for all items). Participants were asked to indicate how often they showed each behavior, relative to given opportunities. Answers were given on a scale from 0 (*never*) to 4 (*always*) and a mean was calculated per construct across related items.

### Analysis

Data analysis was performed using R (Version 4.0.1 [49]) with the packages *psych* [50] and *lavaan* [51]. The level of significance for all analyses was *α* = .05. The suggested bifactorial structure with agency and communion as latent variables was examined for all participants by applying confirmatory factor analyses (CFA) with a maximum likelihood estimator. We used three parcels per factor with five items as manifest variables to account for skewed item distributions [52]. Item allocation to parcels was determined by factor loadings using the item-to-construct balancing approach (for this approach as well as for advantages and disadvantages of item parceling, see [53]). Specifically, the three items with highest loadings were sorted to one parcel each and items with the next highest loadings were subsequently assigned in an inverted order until no items remained. This way each parcel consists of items with approximately equal factor loadings. Presented correlations are Pearson’s product moment correlation coefficients that were computed for all participants in case of relations between SJTs and the science subtest or for the subsample participating in the online study in case of relations to self-reported personality and past behavior. Interpretation of correlations follows a guideline for effect sizes in individual differences research that proposes .10 as relatively small, .20 as medium, and .30 as relatively large [38]. We additionally computed two multiple linear regressions in which agentic or communal behavior were entered as outcomes and the two SJT scales as simultaneous predictors.

## Results

### Dimensionality

**Fig. 3:**
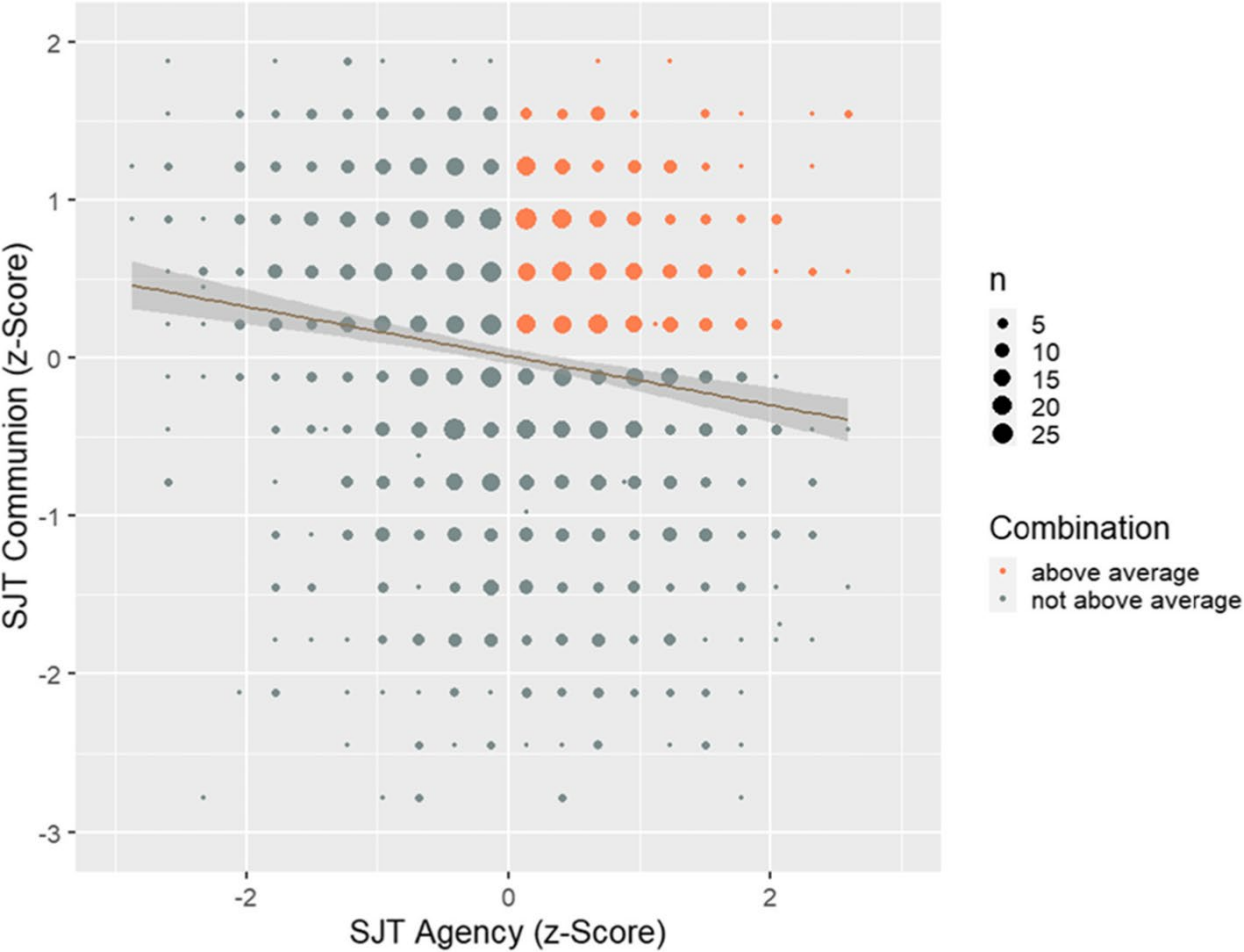
Distribution and relation of construct-driven Situational Judgement Tests Scales. Combination reflects whether a participant‘s construct-driven SJT scores are above average, i.e., whether a participant possesses high skills in agency *and* communion, which is the case for 363 of 1527 participants (24%). The grey markings around the regression line indicate the 95% confidence interval (*b* = −.17, 95% CI [−.22; −.12], *p* < .001)

A two-factor model with correlated latent factors (*r* = −.31; see Fig. 3 for a scatter plot of the relation between both manifest variables) yielded good model fit (χ^2^ (8) = 14.96, CFI = .99, RMSEA = .02, SRMR = .02; see Table 2 and Supplement B and C for more information). This model fitted the data significantly better than a one-factor model (Δχ^2^ (1) = 246.33, *p* < .001) or a two-factor model with uncorrelated latent factors (Δχ^2^ (1) = 45.53, *p* < .001). Reliability analyses revealed Cronbach’s Alpha of α = .63 for agency and α = .70 for communion, respectively.

**Table 2:** Model fit and model comparison

Model	CFI	RMESA	RMSEA 90% CI	SRMR	AIC	BIC	*χ*^*2*^	*df*	*p*	*Δχ*^*2*a^
One factor	.68	.14	[.12, .15]	.09	3462.91	3526.79	261.29	9	< .001	246.33^***^
Two factors (uncorrelated)	.94	.06	[.05, .08]	.06	3262.11	3325.99	60.49	9	< .001	45.53^***^
Two factors (correlated)	.99	.02	[.00, .04]	.02	3218.58	3287.79	14.96	8	< .001	–

*CFI* Comparative Fit Index, *RMESA* Root Mean Square Error of Approximation, *SRMR* Standardized Root Mean Square Residual, *AIC* Akaike Information Criterion, *BIC* Bayesian Information Criterion. ^a^Compared to the two factors correlated model. ***p < .001. *N* = 1527

### Convergent and discriminant validity

Higher scores in SJT Agency were related to higher scores in the traditional HAM-SJT (*r* = .08, 95% CI [.03, .13], *p* < .01; see Table 1) and the science subtest of the HAM-Nat (*r* = .10, 95% CI [.05, .15], *p* < .001) 4. SJT Agency was positively related to self-reported agentic personality (*r* = .10, 95% CI [.01, .18], *p* = .021) and agentic past behavior (*r* = .10, 95% CI [.02; .18], *p* = .017), however effect sizes were small and slightly weaker than hypothesized (*r* = .20) as the confidence intervals exclude this value. Unexpectedly, SJT Agency correlated negatively with self-reported communal personality (*r* = −.14, 95% CI [−.22, −.06], *p* < .001) and self-reported communal past behavior (*r* = −.10, 95% CI [−.18; −.02], *p* = .016). When simultaneously controlling for SJT Communion, SJT Agency predicted agentic (*β* = .09, *p* = .039) but not communal behavior (*β* = −.08, *p* = .078; see Supplement E for more details).

Higher scores in SJT Communion were associated with higher scores in the HAM- SJT (*r* = .23, 95% CI [.19, .28], *p* < .001) but were unrelated to science subtest scores (*r* = .03, 95% CI [−.02, .08], *p* = .229). As expected, SJT Communion was positively related to self-reported communal personality (*r* = .15, 95% CI [.07, .23], *p* < .001) and communal past behavior (*r* = .12, 95% CI [.04; .20], *p* < .01) with the coefficient for self-reported communal personality ranging within the hypothesized magnitude. SJT Communion could predict communal behavior (*β* = .11, *p* = .013), when controlling for SJT Agency.

## Discussion

The aim of this study was to investigate whether a construct-driven SJT can overcome construct validity issues of traditional SJTs in a medical admission context. Construct-driven SJTs differ from traditional SJTs as their development and scoring is guided by constructs defined in advance. We developed an SJT measuring agency and communion and validated it within an actual high-stakes admission process with non-preselected applicants.

While traditional SJTs mostly display one general and undifferentiated factor, we were able to depict agency and communion as two distinct but correlated factors. Reliability of both scales was acceptable and associations to related self-reported personality measures appeared mostly as expected although partly smaller in size. Moreover, we observed small predictions of agency for self-reported past agentic behavior and of communion for past communal behavior regardless of reciprocal control.

### Relations to personality and admission tests

Relations of the SJT to self-reported personality scales were significant, yet generally smaller than expected. One reason can be the different concepts measured by the SJT compared to the self-reported personality tests, i.e., skills versus general personality. That is, in the SJT we aimed to assess social skills which include knowledge and ability aspects and refer to one’s capacity to show specific behaviors with respect to situational requirements (maximum performance). Personality represents general cognitive, affective, and behavioral tendencies (typical performance). For example, a generally reserved person (i.e., low agency as a personality trait) might show agentic behavior at times if necessary in a given situation (i.e., high agency as a social skill [4, 54]). As we collected SJT data under high-stakes conditions, and personality test data under low-stakes conditions in a voluntary survey, the difference between maximum and typical performance may have been exacerbated. Consequently, the small relations between SJT scales and personality tests underpin the possibility that both methods measure different concepts. Another reason that can diminish the relation magnitude is the level of contextualization. While our SJT was contextualized to situations happening within the everyday life of a medical student, personality tests were non-contextualized. As a result, participants might create individual contexts around the personality test items and the frame-of-reference differs from the medical context of the SJT items [55].

The positive correlation between the construct-driven and traditional SJT seems reasonable as both tests aimed at inferring social skills with the same method under comparable conditions. The higher relation of the traditional SJT to the communion scale can be explained by drawing on prior research on traditional SJTs that emphasizes the role of prosocial behavior [56]. In sum, the construct-driven SJT shows small relations to other admission tests, yet offers enough own variance that warrants its usage as a different assessment method.

### Selecting medical students with construct-driven SJTs

Most importantly, construct-driven SJTs allow faculties to define desirable skills representing their profile and select applicants accordingly. Their application might be especially interesting for undergraduate selection where no job-specific skills are expected from applicants but rather general social skills are of interests. How construct scores finally influence selection decision might vary across faculties. One example for a screening method is to combine scores of agency and communion and define scores above average as beneficial. This criterion applied to about one fourth of the present sample that showed the desired combination of agency and communion above average (see Fig. 3). Faculties could also use scores linear and distinguish more fine-graded between applicants or weight the two constructs differently according to their selection strategy or profile. As another point to consider, first results indicate construct-driven SJTs to be less susceptible to faking than personality tests, thus providing a more reliable picture of participants [57]. In contrast to complex admission methods that assess applicants’ actual behavior like MMIs [58], SJTs are more economical to administer and can be completed by a high number of applicants simultaneously.

However, the application of construct-driven SJTs also yields some possible challenges in high-stakes situations. Construct-driven SJTs might still be prone to faking, especially when applicants and coaching agencies become aware of addressed constructs. As constructs are unlikely to change rapidly across years, the identification of crucial constructs becomes easier for outsiders. Moreover, with all forms of SJTs applicants indicate hypothetical rather than actual behavior. This makes SJTs a suitable economic screening method which can then be complemented by more complex methods that assess actual (construct-relevant) behaviors like MMIs [36].

### Limitations and future research

First, we focused on agency and communion as two core skills for future physicians. Nevertheless, we do not assume that they are the only relevant skills nor that they are equally relevant for all medical disciplines. We encourage future research to create construct-driven SJTs inferring other skills. Another important skill that is often mentioned in addition to agency and communion is emotional stability or resilience [59, 60]. In fact, the assessment of resilience within an SJT might be more difficult as the construct is mainly characterized by internal processes or physiological responses which are not directly observable. Generally, items need to be carefully created which renders the process resource-intensive but should ultimately yield payoffs in the form of higher validity.

Second, we showed that SJT scales were associated with relevant self-reported past behavior. Although these relations are first indicators for criterion validity, they do not replace a robust criterion analysis of applicants’ future behavior. Further research should relate construct-driven SJT scores to future behavior as provided, for example, by observations of raters within objective structured clinical examinations (OSCEs) or by ratings of supervisors within everyday work samples. Ideally, future behavior ratings should focus on agentic (e.g., how assertive is the student facing a non-compliant colleague) and communal behavior (e.g., how warm is the student facing a despondent patient), thereby offering a good predictor-criterion fit [61]. Regarding the present example, it would be especially interesting whether the identified subsample of participants with high SJT scores on agency and communion (see Fig. 3) provide better results on criterion measures.

## Conclusion

The present study is the first to demonstrate indicators of validity for a construct-driven SJT assessing agency and communion in medical school applicants. Findings suggest that the construct-driven approach can overcome limitations of traditional SJTs that often struggle with dimensionality and interpretability of scores. However, application in high-stakes contexts might also be accompanied by possible challenges such as prevention of faking and transfer to actual behavior. We encourage further research to collect more robust evidence on the validity of construct-driven SJTs and their application under high-stakes conditions.

## Supplementary Information

**Additional file 1. **Online supplement with more detailed information of the construct-driven SJT development process, item statistics, and results.

## Data Availability

Data that support the findings of this study are available from the stav but restrictions apply to the availability of data so they are not publicly available. Data are however available via kontakt@projekt-stav.de upon reasonable request and with permission of the stav. Additional material including a preregistration, R code, the data request, and the online supplement can be retrieved from the Open Science Framework via https://osf.io/6579b/.

## Abbreviations

SJT: Situational Judgment Test
MMI: Multiple Mini Interview
BFI-2: Big Five Inventory 2
IAL: Interpersonale Adjektivliste [Interpersonal adjective list]
ISK: Inventar sozialer Kompetenzen [Inventory of social competencies]
CFA: Confirmatory Factor Analysis
CFI: Comparative Fit Index
RMSEA: Root Mean Square Error of Approximation
SRMR: Standardized Root Mean Square Residual
CI: Confidence Interval
OSCE: Objective Structured Clinical Examination

## Footnotes

1 One applicant answered less than 80% of the SJT Agency items and, as defined in the preregistration, was excluded for analyses regarding this scale.

2 We did not include an a-priori power analysis in our preregistration. However, a sensitivity analysis performed with G*Power [39] revealed that with our sample of *N* = 575 we are able to reliably detect even small effect sizes of 0.10 with a power of 80% (alpha = .05, one-tailed).

3 Emotional stability (or resilience) was proposed as a third important construct for physicians. We developed and tested SJT items on emotional stability, but after their discriminant validity in the pilot study was extremely poor, we decided to concentrate only on agency and communion in the following.

4 Both scales were unrelated to high-school GPA (SJT Agency: *r* = -.03, 95% CI [-.09; .02], *p* = .203; SJT Communion: *r* = .02, 95% CI [-.03; .08], *p* = .424) and showed small relations to two reasoning tests, i.e., arithmetic problem solving (SJT Agentic: *r* = .08, 95% CI [.03; .13], *p* < .01; SJT Communion: *r* = .07, 95% CI [.02; .12], *p* < .01) and logical reasoning (SJT Agentic: *r* = .10, 95% CI [.05; .15], *p* < .001; SJT Communion: *r* = .10, 95% CI [.05; .15], *p* < .001). Data for the third reasoning test has not been available for the present sample yet.
