# Supplementary material for: Assessing distinguishable social skills in medical admission: does construct-driven development solve validity issues of situational judgment tests?
Source: BMC Med Educ. 2022 Apr 20;22:293. doi: 10.1186/s12909-022-03305-x (PMC9020047; doi:10.1186/s12909-022-03305-x)
Supplement: Supplementary file 1 — Additional file 1. Online supplement with more detailed information of the construct-driven SJT development process, item statistics, and results. [file 12909_2022_3305_MOESM1_ESM.pdf]

Assessing Distinguishable Social Skills in Medical Admission - Does Construct-Driven Development Solve Validity Issues of Situational Judgment Tests?

Ina Mielke[1], Simon M. Breil[2], Dorothee Amelung[3], Lia Espe[4], & Mirjana Knorr[1]

[1]Department of Biochemistry and Molecular Cell Biology, University Medical Center Hamburg-Eppendorf, Germany

[2]Department of Psychology, University of Münster, Germany

[3]Office of Student Affairs, Heidelberg University Hospital, Germany

[4]Division of Medical Teaching and Education Research, Göttingen University Medical Center, Germany

Supplemental Material A

Detailed Description of the Construct-Driven SJT Development Process

The development of the Situational Judgment Test (SJT) started with a workshop with an SJT-expert and researchers from several medical faculties. Within the workshop, a summary of earlier traditional SJT items was discussed and the development of construct-driven SJT items was trained. Based on these insights and material, researchers from the same medical faculties developed SJT situations (*N* =229) and related behavioral responses (four to nine per situation) that aimed at measuring agency, communion, or emotional stability. After another critical review, a total of 171 SJT items were selected and each rated by two other independent construct experts. The experts rated the SJT items according to the intended construct and the behavioral responses according to the level of the construct. If the experts agreed on the intended construct and on the construct level of at least three behavioral responses (every behavior measuring another construct level), this SJT item was selected for a pretest study. A total of 60 SJT situations with three behavioral responses that reflected three levels of the construct (i.e., low, medium, high) resulted. The item set included of 20 items per construct.

The pretest was an online survey that was completed by undergraduate psychology students from two German universities (*N* = 54; 91% female) with an age ranging from 18 to 48 years (*M* = 23.02, *SD* = 5.66). The survey included the 60 SJT items and a Big Five questionnaire (1). The results showed promising reliability and validity (see Table A1).

For the following pilot study, 15 SJT items per construct were selected based on their discriminatory power. The pilot study was done as a voluntary study after the HAM-Nat within the admission process for medical studies at the medical faculty in Hamburg and was started by 912 participants. We excluded 76 participants as they completed less than the predefined 80% of the items in one of the construct-driven SJT scales. The final sample consisted of 836 participants (61% female, 30% male, 8% no answer) with a mean age of 21.27 years (range: 18-36, *SD* = 2.00, 8% no answer). Those participants who agreed to be contacted for further studies after the admission process received an invitation to an additional online survey two weeks later. This online survey included personality questionnaires to assess the Big Five (BFI; 1), social competencies (ISK; 2), and interpersonal behavior (IAL; 3) and was completed by 253 participants. The results of the pilot study were different

from the pretest study with lower reliabilities and lower validity (see Table A2). Especially emotional stability showed low reliability and no discriminant validity.

Based on these results, we decided to concentrate on agency and communion and started an intense review process. In a first step, items were again critically revised by the authors to ensure good construct-fit. Next, two personality psychologists who had not been involved in earlier development steps were asked to match the situations to one of six constructs. The six constructs were facets of the Big Five traits extraversion (assertiveness and energy level), agreeableness (compassion and respectfulness), and emotional stability (anxiety and depression). If the selected construct was not the intended one (i.e., either assertiveness for agency or compassion for communion), the situation was edited accordingly. We then checked whether each situation included an appropriate dilemma which would (a) prevent participants from identifying the behavioral response exhibiting the most optimal expression of the intended construct while (b) at the same time not reflecting expressions of the other construct (e.g., an agency item should not include a dilemma that triggers communion). In a next step, four to seven behavioral responses per situation were developed and rated by three personality psychologists according to their construct level. Finally, three behavioral responses per item were selected based upon similar ratings and representation of the three different levels of the construct. The resulting SJT consisted of 15 situations per construct with three behavioral responses reflecting each either a low, medium, or high level of the construct.

Table A1

Descriptive Statistic, Reliability, Convergent and Discriminant Validity Pretest Study

Test	Variable	*M (SD)*	*α*	1.	2.	3.	4.	5.	6.	7.	8.	9.	10.
SJT	1.Agency	2.42 (0.26)	.78	-									
	2.Communion	2.47 (0.26)	.82	.01	-								
	3.Emotional Stability	2.33 (0.32)	.78	**.47**	**.28**	-							
BFI	4.Extraversion	3.49 (0.63)	.88	**.41**	.24	**.33**	-						
	5.Assertiveness	3.36 (0.77)	.83	**.48**	.00	.23	**.77**	-					
	6.Agreeableness	3.88 (0.65)	.91	-.04	**.51**	.19	**.31**	.10	-				
	7.Compassion	4.07 (0.79)	.89	-.08	**.50**	.09	**.28**	.13	**.91**	-			
	8.Neuroticism	2.75 (0.76)	.93	-.15	**-.39**	**-.49**	**-.48**	**-.38**	**-.57**	**-.34**	-		
	9.Anxiety	3.13 (0.74)	.75	-.07	**-.27**	**-.43**	**-.36**	**-.30**	**-.40**	-.21	**.91**	-	
	10.Conscientiousness	3.50 (0.64)	.86	.25	**.34**	**.30**	**.41**	**.39**	**.52**	**.44**	**-.55**	**-.44**	-
	11.Openness	3.77 (0.72)	.91	.01	.13	.23	**.29**	**.33**	**.42**	**.51**	-.25	-.17	.22

Note. Bold coefficients are significant with $p < .05$. Framed fields indicate hypothesized relations and significant relations here are in the hypothesized direction. Indented variables are selected BFI facets. SJT = Situational Judgment Test, BFI = Big Five Inventory (1).

Table A2

Descriptive Statistic, Reliability, Convergent and Discriminant Validity Pilot Study

Test	Variable	*M (SD)*	*α*	1.	2.	3.	4.	5.	6.	7.	8.	9.	10.	11.	12.	13.	14.	15.	16.	17.	18.	19.	20.	21.
SJT	1. Agency	2.45 (0.21)	.59	-																				
	2. Communion	2.52 (0.22)	.74	-.04	-																			
	3. Emotional Stability	2.46 (0.19)	.50	**.22**	**.17**	**-**																		
	4. Traditional	3.48 (0.15)	.70 [a]	**.15**	**.23**	**.11**	**-**																	
	5. HAM Nat Science Test	27.25 (9.52)	-[b]	**.07**	-.06	.01	.03	-																
BFI	6. Extraversion	3.69 (0.59)	.84	**.15**	.05	**.29**	.10	.04	-															
	7. Assertiveness	3.67 (0.71)	.78	**.17**	-.05	**.22**	.05	.04	**.74**	-														
	8. Agreeableness	4.15 (0.43)	.78	-.03	**.32**	**.22**	.10	-.11	**.16**	-.09	-													
	9. Compassion	4.47 (0.51)	.69	.02	**.26**	**.23**	.12	**-.17**	**.30**	.08	**.76**	-												
	10.Neuroticism	2.29 (0.59)	.86	-.03	-.07	**-.28**	-.01	-.09	**-.29**	**-.18**	**-.47**	**-.30**	-											
	11.Anxiety	2.63 (0.69)	.64	-.01	-.05	**-.24**	-.03	-.05	**-.27**	**-.22**	**-.28**	**-.16**	**.86**	-										
IAL	12. Assured-Dominant	5.48 (1.09)	.70	**.14**	-.07	**.16**	-.01	.11	**.58**	**.68**	**-.19**	-.06	**-.19**	**-.23**	-									
	13. Unassured-Submissive	3.56 (1.37)	.75	**-.17**	-.02	**-.24**	-.08	-.08	**-.78**	**-.55**	**-.13**	**-.17**	**.34**	**.34**	**-.47**	-								
	14. Warm-Agreeable	6.83 (0.92)	.69	-.02	**.25**	**.14**	.07	**-.16**	**.21**	.04	**.55**	**.68**	**-.16**	-.02	-.01	-.02	-							
	15. Cold-Hearted	1.92 (0.97)	.72	-.03	**-.15**	**-.22**	**-.15**	.09	**-.24**	-.03	**-.58**	**-.63**	**.31**	.12	.12	**.28**	**-.44**	-						
	16. Neurotic	3.63 (1.00)	.58	-.01	-.04	**-.22**	-.06	-.07	**-.21**	**-.22**	**-.21**	-.07	**.68**	**.75**	**-.20**	**.33**	.02	**.19**	-					
ISK	17. Agency	66.94 (9.31)	.86	**.17**	-.01	**.24**	.03	.10	**.75**	**.71**	.04	**.15**	**-.36**	**-.37**	**.62**	**-.70**	.09	-.10	**-.35**	-				
	18. Assertiveness	19.84 (3.18)	.76	**.18**	-.08	**.15**	.05	**.16**	**.53**	**.74**	**-.18**	-.06	**-.17**	**-.20**	**.66**	**-.47**	-.06	.11	**-.22**	**.77**	-			
	19. Social Orientation	103.65 (10.09)	.88	-.04	**.24**	**.23**	.06	-.10	**.25**	.06	**.69**	**.70**	**-.46**	**-.31**	-.07	**-.20**	**.56**	**-.54**	**-.25**	**.26**	.00	-		
	20. Prosociality	23.67 (2.71)	.67	-.02	**.26**	**.26**	**.13**	**-.14**	**.27**	.09	**.60**	**.65**	**-.35**	**-.24**	-.02	**-.23**	**.48**	**-.56**	**-.17**	**.24**	.04	**.79**	-	
	21. Self Regulation	81.56 (8.48)	.83	.07	.04	**.25**	-.01	.11	**.22**	**.25**	**.34**	**.20**	**-.72**	**-.59**	**.17**	**-.28**	.11	**-.17**	**-.57**	**.37**	**.30**	**.42**	**.31**	-
	22. Emotional Stabiliy	16.77 (3.14)	.71	-.02	.09	**.20**	-.04	.08	.08	.05	**.34**	**.17**	**-.69**	**-.55**	.04	**-.13**	.09	-.08	**-.48**	**.20**	.12	**.34**	**.25**	**.75**

Note. Bold coefficients are significant with $p < .05$. Framed fields indicate hypothesized relations and significant relations here are in the hypothesized direction. Indented variables are selected facets. SJT = Situational Judgment Test, BFI = Big Five Inventory, IAL = Interpersonal Adjective List, ISK = Inventory of Social Competencies. [a] Reliability was computed for a sample of $N = 916$ where no control for missing construct-driven SJT items was applied. [b] Single item values per participant were not available but internal consistency for the test usually tends to be acceptable (4).

Supplemental Material B

Detailed Item Information Construct-Driven SJT

Table B1

Construct-Driven SJT Item Statistics

				Distribution			Loadings	
Scale	Item	M	SD	Median	Skew	Kurtosis	Model 1	Model 2
Agency	A1	1.25	0.56	1	2.14	3.45	0.35	
	A2	1.74	0.69	2	0.40	-0.90	0.27	
	A3	2.33	0.52	2	0.23	-0.87	0.34	
	A4	2.33	0.79	3	-0.66	-1.08	0.34	
	A5	1.81	0.62	2	0.15	-0.55	0.34	
	A6	2.02	0.85	2	-0.03	-1.62	0.40	
	A7	2.49	0.52	2	-0.17	-1.46	0.51	
	A8	1.97	0.61	2	0.02	-0.33	0.22	
	A9	1.74	0.58	2	0.10	-0.49	0.47	
	A10	1.69	0.54	2	-0.09	-0.68	0.33	
	A11	2.34	0.68	2	-0.54	-0.78	0.52	
	A12	2.66	0.61	3	-1.62	1.41	0.10	
	A13	2.24	0.95	3	-0.48	-1.71	0.17	
	A14	1.87	0.98	1	0.26	-1.90	0.18	
	A15	2.04	0.43	2	0.20	2.29	0.33	
Communion	C1	2.83	0.44	3	-2.56	6.02		0.30
	C2	2.80	0.43	3	-2.00	3.18		0.40
	C3	2.55	0.60	3	-0.96	-0.10		0.40
	C4	2.88	0.39	3	-3.36	11.06		0.35
	C5	2.38	0.50	2	0.31	-1.44		0.39
	C6	2.86	0.38	3	-2.86	7.89		0.19
	C7	2.45	0.65	3	-0.77	-0.48		0.21
	C8	2.71	0.54	3	-1.73	2.05		0.33
	C9	2.62	0.62	3	-1.41	0.83		0.44
	C10	2.79	0.46	3	-2.20	4.16		0.35
	C11	2.66	0.58	3	-1.54	1.31		0.55
	C12	2.61	0.66	3	-1.43	0.68		0.43
	C13	2.00	0.42	2	0.03	2.54		0.36
	C14	2.73	0.48	3	-1.46	1.08		0.46
	C15	2.49	0.68	3	-0.96	-0.30		0.41

Note. Standardized loadings from CFA with one-factor models, where each item loads on the respective latent factor and manifest and latent variables are standardized.

Supplemental Material C

Results Confirmatory Analyses

Figure C1

Two Factors Correlated Model

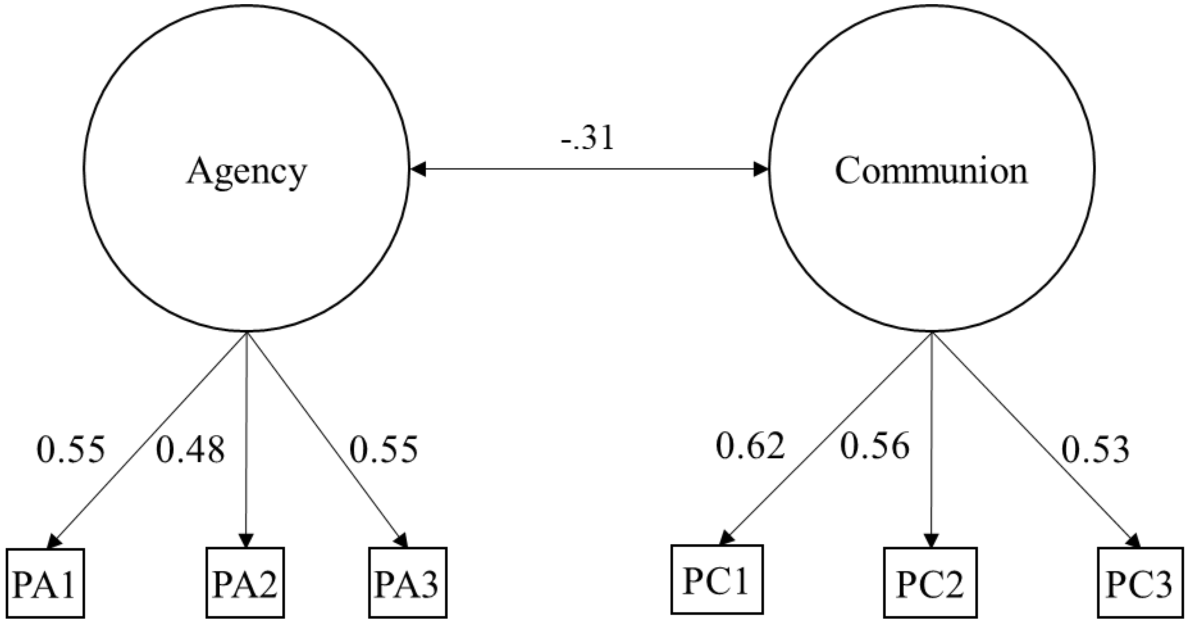

Note. Manifest variables are parcels of three items. Values per parcel are standardized estimates for which the latent variables and the manifest variables had been standardized.

Supplemental Material D

Correlations Between SJT Scales and all Personality Measures

Table D1

Reliability and Correlations of SJT Scores and Personality Measures (Extension of Table 2 From the Main Text)

Test	Variable	Rel	1.	2.	3.	4.	5.	6.	7.	8.	9.	10.	11.	12.	13.	14.	15.	16.	17.	18.	19.	20.	21.	22.	23.	24.	25.	26.	27.	28.	29.	30.	31.	32.	33.
SJT	1.Agency	.63	-																															
	2.Communion	.70	**-.17**	-																													
BFI	3.Extraversion	.84	.04	-.02	-																												
	4.Sociability	.81	.01	-.03	**.88**	-																											
	5.*Assertiveness*	.73	**.08**	-.01	**.73**	**.46**	-																										
	6.Energy Level	.69	.00	.00	**.78**	**.57**	**.32**	-																									
	7.Agreeableness	.74	**-.14**	**.14**	**.16**	**.15**	-.07	**.28**	-																								
	8.*Compassion*	.63	**-.14**	**.15**	**.18**	**.16**	.00	**.27**	**.78**	-																							
	9.Respect	.61	**-.10**	.08	.07	.02	-.02	**.18**	**.73**	**.48**	-																						
	10.Trust	.56	-.08	**.10**	**.10**	**.16**	**-.13**	**.19**	**.78**	**.36**	**.29**	-																					
	11.Neuroticism	.84	.01	.02	**-.35**	**-.28**	**-.27**	**-.30**	**-.30**	**-.16**	**-.33**	**-.21**	-																				
	12.Conscientiousness	.86	.02	.07	**.22**	**.11**	**.19**	**.25**	**.37**	**.34**	**.45**	**.11**	**-.39**	-																			
	13.Openness	.83	-.01	.04	**.37**	**.24**	**.29**	**.38**	**.21**	**.22**	**.15**	**.11**	**-.22**	**.19**	-																		
IAL	14.*Assured-Dominant*	.71	.07	-.05	**.57**	**.46**	**.65**	**.27**	**-.19**	-.07	**-.15**	**-.20**	**-.31**	**.14**	**.24**	-																	
	15.*Unassured-Submissive*	.72	-.05	-.02	**-.72**	**-.75**	**-.55**	**-.40**	-.01	-.05	**.08**	-.04	**.33**	**-.10**	**-.24**	**-.55**	-																
	16.*Warm-Agreeable*	.76	**-.14**	**.10**	**.15**	**.15**	-.03	**.25**	**.54**	**.56**	**.41**	**.30**	**-.12**	**.26**	**.21**	**.09**	-.03	-															
	17.*Cold-Hearted*	.74	.05	**-.11**	**-.23**	**-.20**	-.06	**-.27**	**-.46**	**-.51**	**-.33**	**-.24**	**.18**	**-.31**	**-.23**	**-.11**	**.16**	**-.69**	-														
	18.Unassuming-Ingenuous	.63	**-.14**	.00	**-.25**	**-.19**	**-.31**	**-.10**	**.31**	**.25**	**.29**	**.19**	.03	**.20**	-.06	**-.28**	**.37**	**.35**	**-.15**	-													
	19.Aloof-Introverted	.68	.01	-.04	**-.46**	**-.47**	**-.19**	**-.42**	**-.34**	**-.30**	**-.22**	**-.26**	**.31**	**-.25**	**-.18**	**-.24**	**.44**	**-.40**	**.50**	.02	-												
	20.Arrogant-Calculating	.70	**.10**	-.07	.04	.04	**.15**	**-.09**	**-.44**	**-.33**	**-.43**	**-.28**	**.20**	**-.36**	**-.09**	**.19**	**-.11**	**-.45**	**.50**	**-.31**	**.31**	-											
	21.Gregarious-Extraverted	.80	-.04	.02	**.66**	**.68**	**.33**	**.53**	**.33**	**.32**	**.18**	**.25**	**-.34**	**.20**	**.25**	**.43**	**-.54**	**.50**	**-.46**	.02	**-.61**	**-.15**	-										
ISK	22.Agency	.87	**.09**	-.02	**.68**	**.59**	**.61**	**.42**	.02	.04	.04	-.02	**-.48**	**.28**	**.35**	**.63**	**-.66**	.07	**-.14**	**-.27**	**-.32**	**.11**	**.50**	-									
	23.*Assertiveness*	.76	**.11**	.00	**.48**	**.32**	**.64**	**.22**	**-.13**	-.07	-.02	**-.18**	**-.25**	**.16**	**.26**	**.58**	**-.46**	-.05	-.04	**-.31**	**-.14**	**.20**	**.22**	**.76**	-								
	24.Conflict Willing	.70	**.11**	-.02	**.29**	**.24**	**.33**	**.14**	**-.16**	**-.13**	**-.18**	-.08	**-.18**	**-.09**	**.24**	**.36**	**-.35**	**-.16**	.07	**-.34**	-.02	**.27**	**.12**	**.62**	**.45**	-							
	25.Extraversion	.86	.01	-.05	**.72**	**.75**	**.42**	**.52**	**.20**	**.16**	**.10**	**.18**	**-.38**	**.19**	**.26**	**.45**	**-.63**	**.20**	**-.21**	**-.10**	**-.46**	-.02	**.69**	**.73**	**.37**	**.19**	-						
	26.Decisionmaking	.81	.03	.00	**.36**	**.26**	**.37**	**.25**	**.09**	**.09**	**.17**	-.03	**-.51**	**.48**	**.22**	**.41**	**-.39**	**.12**	**-.16**	-.06	**-.23**	-.08	**.29**	**.72**	**.41**	**.25**	**.35**	-						

Table D1 (continued).

	27.Social Orientation	.89	**-.10**	**.12**	**.28**	**.26**	.08	**.33**	**.60**	**.56**	**.47**	**.37**	**-.37**	**.38**	**.37**	.07	**-.15**	**.47**	**-.45**	**.18**	**-.39**	**-.37**	**.37**	**.32**	.06	.08	**.34**	**.37**	-							
	28.*Prosociality*	.66	**-.12**	**.11**	**.25**	**.25**	.03	**.30**	**.55**	**.56**	**.39**	**.33**	**-.28**	**.31**	**.24**	.03	**-.13**	**.46**	**-.45**	**.14**	**-.42**	**-.30**	**.35**	**.21**	.00	.00	**.29**	**.23**	**.77**	-						
	29.Perspective Change	.79	-.08	**.11**	**.16**	**.12**	.06	**.21**	**.47**	**.48**	**.40**	**.24**	**-.23**	**.27**	**.30**	.02	-.06	**.42**	**-.35**	**.16**	**-.24**	**-.26**	**.25**	**.22**	.07	.07	**.19**	**.28**	**.76**	**.47**	-					
	30.Value Pluralism	.67	-.02	.03	**.22**	**.21**	.07	**.25**	**.42**	**.35**	**.35**	**.28**	**-.36**	**.25**	**.35**	**.11**	**-.13**	**.35**	**-.29**	**.14**	**-.24**	**-.27**	**.28**	**.35**	**.10**	**.16**	**.33**	**.35**	**.74**	**.45**	**.54**	-				
	31.Compromise Willing	.76	**-.08**	.04	**.14**	**.12**	.03	**.18**	**.39**	**.37**	**.30**	**.24**	**-.17**	**.21**	**.21**	-.01	-.01	**.31**	**-.24**	**.20**	**-.17**	**-.26**	**.19**	**.11**	-.03	-.03	**.16**	**.16**	**.72**	**.45**	**.47**	**.44**	-			
	32.Listening	.80	-.06	**.14**	**.25**	**.23**	**.11**	**.26**	**.38**	**.31**	**.30**	**.26**	**-.32**	**.35**	**.27**	**.10**	**-.22**	**.23**	**-.33**	.03	**-.36**	**-.27**	**.29**	**.29**	.08	**.09**	**.26**	**.34**	**.69**	**.47**	**.35**	**.32**	**.31**	**-**		
NQ	33. Admiration	.71	.00	-.05	**.22**	**.12**	**.26**	**.16**	**-.09**	**-.09**	**-.08**	-.06	.01	-.02	**.12**	**.22**	**-.14**	-.06	**.14**	-.06	.02	**.16**	**.13**	**.23**	**.29**	**.10**	**.20**	.06	**-.11**	**-.16**	-.06	.00	**-.09**	**-.10**	**-**	
	34. Rivalry	.62	.07	**-.10**	-.08	**-.10**	**.09**	**-.18**	**-.40**	**-.36**	**-.33**	**-.26**	**.24**	**-.32**	**-.21**	.05	.02	**-.37**	**.41**	**-.19**	**.29**	**.44**	**-.21**	-.05	**.09**	.07	**-.11**	**-.14**	**-.48**	**-.41**	**-.27**	**-.32**	**-.36**	**-.38**	**.33**

Note. Italic variables were aggregated into one composite personality score for agency or communion and grey fields indicate their intercorrelations. Framed fields indicate hypothesized relations and significant relations here are in the hypothesized direction. Bold coefficients are significant with $p < .05$. SJT = Situational Judgment Test, ISK = Inventory of Social Competencies, IAL = Interpersonal Adjective List, NQ = Narcissistic Admiration and Rivalry Questionnaire (5).

Supplement Material E

Detailed Information on Self-Reported Past Behavior

Table E1

Self-Reported Past Behavior Items

Construct	Item
	Compared to the opportunities you have had, how often in the last 6 months have you…
Agency	Stood up for your own needs?
	Asserted yourself against others?
	Convinced others of your opinion?
	Addressed a conflict?
Communion	Offered help to others?
	Showed understanding for others’ problems?
	Supported others emotionally?
	Honestly asked about the well-being of others?

Note. Answers were given on a scale from 0 (*never*) to 4 (*always*).

Table E2

Multiple Regression With Agentic and Communal Self-Reported Past Behavior as Criteria

Model	*b*	95% CI	*β*	*p*	R^2	Adj R^2	*F*	*df*	*p*
Agentic Behavior					.01	.01	3.40	571	< .05
SJT Agency	**0.19**	**[0.01; 0.36]**	**0.09**	**.039**					
SJT Communion	-0.13	[-0.36; 0.11]	-0.04	.293					
Communal Behavior					.02	.02	6.03	571	< .05
SJT Agency	-0.15	[-0.31; 0.02]	-0.08	.078					
SJT Communion	**0.28**	**[0.06; 0.49]**	**0.11**	**.013**					

Note. Bold coefficients are significant with $p < .05$. SJT = Situational Judgment Test.

References

1. Danner D, Rammstedt B, Bluemke M, Lechner C, Berres S, Knopf T, et al. Das Big Five Inventar 2. Diagnostica. 2019;65:121-32.
2. Kanning UP. Inventar sozialer Kompetenzen (ISK/ISK-K). Göttingen: Hogrefe; 2009.
3. Jacobs I, Scholl W. IAL-K: Entwicklung einer Kurzform der Interpersonalen Adjektivliste. Diagnostica. 2016;62:227-41.
4. Hissbach JC, Klusmann D, Hampe W. Dimensionality and predictive validity of the HAM-Nat, a test of natural sciences for medical school admission. BMC Med Educ. 2011;11:1-11.
5. Leckelt M, Wetzel E, Gerlach TM, Ackerman RA, Miller JD, Chopik WJ, et al. Validation of the Narcissistic Admiration and Rivalry Questionnaire Short Scale (NARQ-S) in convenience and representative samples. Psychol Assess. 2018;30:86-96.
